# Alzheimer’s disease research must connect vascular molecular phenotypes with physiology

**DOI:** 10.1371/journal.pbio.3004051

**Published:** 2026-10-28

**Authors:** Thomas S. Lewis, Jessica Wagner, Jonas J. Neher

**Affiliations:** 1 Biomedical Center, Metabolic Biochemistry, LMU Medizin, LMU Munich, Munich, Germany; 2 Neuroimmunology and Neurodegenerative Diseases, German Center for Neurodegenerative Diseases (DZNE), Munich, Germany; 3 Department of Cellular Neurology, Hertie Institute for Clinical Brain Research, University of Tübingen, Tübingen, Germany; 4 Munich Cluster for Systems Neurology (SyNergy), Munich, Germany; School of Medicine and Health, Technical University, Munich, GERMANY

## Abstract

Molecular profiling studies have mapped vascular alterations in Alzheimer’s disease, but have not proven a role for them in pathology. This Perspective argues for longitudinal studies to link molecular and structural changes to vascular mechanics, hemodynamics and tissue perfusion.

## Teaser

Molecular profiling studies have mapped vascular alterations in Alzheimer’s disease, but have not proven a role for them in pathology. Longitudinal studies are needed to link molecular and structural changes to vascular mechanics, hemodynamics, and tissue perfusion.

Alzheimer’s disease (AD) research has long focused on how parenchymal disease hallmarks—amyloid-β (Aβ) plaques and tau tangles—drive neurodegeneration. However, growing evidence indicates that vascular dysfunction (i.e., altered vessel mechanics, hemodynamics, neurovascular coupling, tissue perfusion, or oxygen delivery) is a significant and independent contributor to neuronal damage and degeneration. Although blood–brain barrier permeability is also an important aspect of neurovascular physiology, the extent of barrier disruption does not necessarily reflect the presence or severity of vascular dysfunction and has been discussed extensively by others [1]. Therefore, we would argue that distinguishing whether vascular dysfunction causes, amplifies, or merely follows other hallmarks of AD—and identifying the molecular changes that produce functional deficits—is essential for developing earlier diagnostic biomarkers and prioritizing vascular changes as therapeutic targets, rather than correlates of AD.

Neurovascular dysfunction is among the earliest detectable abnormalities in patients with AD and may precede biomarker changes that reflect Aβ and tau pathology [2], with reductions in cerebral blood flow and impaired neurovascular coupling already occurring at prodromal stages of AD (reviewed in [3,4]). At advanced stages of AD, regional cerebral blood flow may decrease by up to 45% in patients [5]. These observations justify treating vascular dysfunction as a potential contributor to disease progression, rather than as a secondary consequence of neuronal pathology. They do not, however, identify the mechanisms that impair vascular function. Recent advances in molecular profiling have enabled the characterization of molecular and cellular changes within cerebral blood vessels [6,7]. However, despite this progress, little is known about the drivers of these alterations, how closely they are linked to protein aggregation, how they progress over the course of disease, or how they translate into measurable changes in vascular function. Longitudinally mapping these molecular and functional changes could enable earlier diagnosis and patient stratification, while also identifying disease stages at which interventions to preserve or restore adequate cerebral perfusion could be tested.

We would argue that technology-driven analysis of molecular and cellular changes in the cerebral vasculature has outpaced our understanding of their functional consequences. Molecular or cellular abnormalities alone cannot establish whether, or to what extent, a vessel is functionally impaired. Closing this gap is now a key challenge for the field that will require directly linking molecular and structural remodeling of the cerebral vasculature to measurements of vascular biomechanics, hemodynamics, tissue perfusion, and oxygen delivery. To enable this advance, we need in vivo models that can be used to establish temporal order and causality. Although genetic mouse models do not recapitulate the full complexity of human AD, they permit vascular alterations to be monitored and manipulated longitudinally and also reproduce age-associated changes, which are of major importance for understanding sporadic AD pathogenesis. These models reproduce selected components of AD pathology, particularly Aβ deposition and cerebral amyloid angiopathy, and, importantly, some also exhibit impaired neurovascular coupling and reduced cerebral blood flow [8,9]. Defining mechanistic links between molecular remodeling and vascular dysfunction in animal models can then guide targeted studies in patients.

Establishing how vascular pathology contributes to AD will require us to determine not only what changes within the vessel wall, but whether these changes alter vascular mechanics, hemodynamics and, ultimately, tissue perfusion. For example, extensive vascular extracellular matrix (ECM) remodeling has been reported in aging and AD, including alterations in collagens, laminins, fibronectin, proteoglycans, and basement membrane proteins [10], many of which can be detected in cerebrospinal fluid and may therefore also serve as biomarkers of vascular pathology [7]. The vascular ECM is more than a structural scaffold; it regulates endothelial signaling, vascular smooth muscle function, basement membrane integrity, arterial compliance, and perivascular clearance. Changes in its composition may therefore have direct consequences for vascular function. For example, changes in collagen organization, elastin integrity, and basement membrane composition may increase arterial stiffness [11]. Moreover, Aβ deposition within vessel walls may impair vessel elasticity, disrupt perivascular clearance and initiate inflammatory cascades. Together, these changes provide a plausible explanation for altered neurovascular function and hypoperfusion in patients with AD [3,4]. However, biological plausibility should not be mistaken for demonstrated physiological dysfunction. The functional consequences of these molecular and structural changes have rarely been measured directly, and vascular dysfunction is therefore frequently inferred from histological or molecular phenotypes rather than quantified physiologically; we believe that this is a critical limitation.

Making the direct link from molecular pathology to vascular dysfunction will require measurements across different spatial and temporal scales, yet no single imaging modality provides all of this information. Two-photon microscopy enables real-time visualization of individual arterioles, capillaries, and vascular cells, revealing mechanisms of neurovascular coupling, vasoconstriction, and vasodilation, but it interrogates only small cortical areas and therefore provides a highly localized view of vascular function [12,13]. By contrast, functional MRI can visualize blood flow throughout the brain, but at substantially lower spatial resolution. Notably, neither approach can determine changes in the passive properties of vessels, such as pulse-wave velocity as a measure of vascular stiffness. High-frequency ultrasound (HFUS) can address this gap by acquiring thousands of frames per second, resolving individual cardiac cycles and enabling estimates of arterial stiffness and vascular resistance (Fig 1). Contrast-enhanced HFUS extends this assessment to microvascular perfusion and computational reconstruction of the functional cerebral vascular network, from meningeal vessels to the microvasculature [14]. HFUS therefore provides a complementary scale between cellular imaging by two-photon microscopy and whole-brain measurements by MRI. However, as no single readout can capture vascular dysfunction at every level, comprehensive analysis will require integration across scales. This is technically demanding because it requires complementary equipment and expertise, as well as anatomical co-registration of datasets from the same animal, often across facilities.

**Fig 1:**
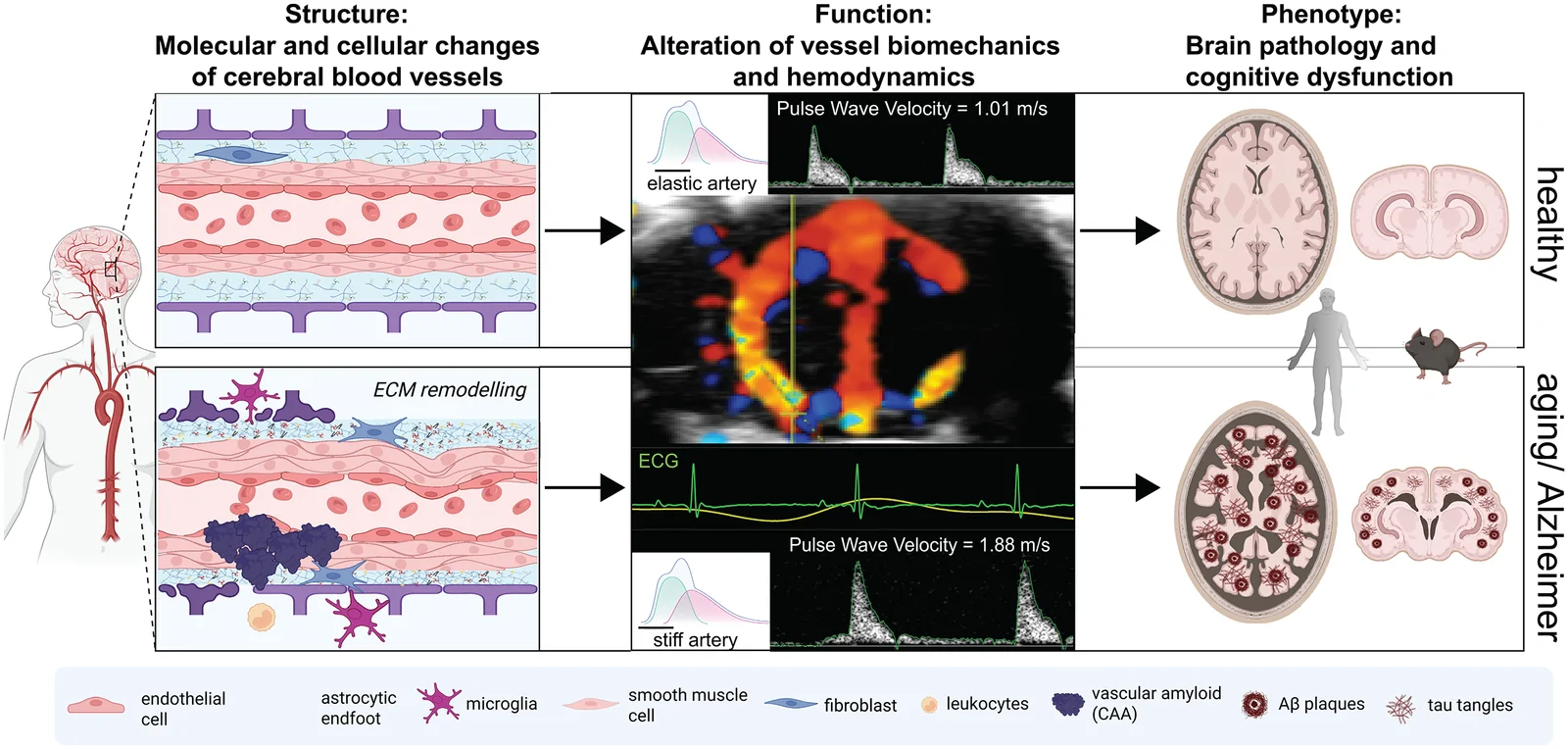
Conceptual framework for improving our understanding of vascular dysfunction in brain aging and Alzheimer’s disease. Future research should aim to delineate the relationship among molecular alterations (e.g., vascular extracellular matrix remodeling, amyloidosis, and inflammation; left panel), altered vessel properties and dysfunction (e.g., arterial stiffening, measured by pulse-wave velocity; middle panel), and brain pathology and cognitive decline (right panel). Figure created in BioRender. Wagner, J. (2026) https://BioRender.com/qxme5vm.

The field has made remarkable progress in defining the molecular and cellular landscape of aged and diseased cerebral vasculature. We see the next major step as delineating how these molecular alterations relate to vascular physiology. Cellular responses, vascular biomechanics, network hemodynamics, tissue perfusion, and oxygenation capture distinct but interconnected aspects of vascular function. Combining measurements across these scales will therefore be essential for determining how molecular and structural vascular changes alter vascular physiology, brain perfusion, and function. Modern imaging techniques now allow detailed characterization of vascular phenotypes in preclinical studies and, for some modalities, in clinical studies. Complemented by histological and spatial omics approaches, these modalities can provide a comprehensive map of where and how blood vessels are affected, how these changes alter vascular network function and how they affect brain homeostasis. Such an approach could be used to identify earlier disease biomarkers, strengthen mechanistic understanding of vascular contributions to neurodegeneration and reveal therapeutic targets, while enabling spatial and temporal monitoring of interventions.

Although we have focused on AD, this framework may also be relevant to other neurodegenerative diseases and to healthy aging. Understanding what changes occur within the brain’s vessels during aging or neurodegeneration is therefore no longer sufficient; we must determine how these changes affect vascular function and brain perfusion, and which components can be targeted to preserve or restore brain health.
